# A Survey of Primary Care Offices: Triage of Poisoning Calls without a Poison Control Center

**DOI:** 10.1155/2012/417823

**Published:** 2012-07-01

**Authors:** Travis Austin, Daniel E. Brooks, Sharyn Welch, Frank LoVecchio

**Affiliations:** ^1^University of Arizona College of Medicine, Phoenix, AZ 85004, USA; ^2^Department of Medical Toxicology, Banner Good Samaritan Medical Center, 925 East McDowell Road, 2nd Floor, Phoenix, AZ 85006, USA; ^3^Banner Good Samaritan Poison and Drug Information Center, Phoenix, AZ 85006, USA

## Abstract

Poison control centers hold great potential for saving health care resources particularly by preventing unnecessary medical utilization. We developed a four-question survey with three poisoning-related scenarios, based on common calls to our poison center, and one question regarding after-hours calls. We identified primary care provider offices in our poison center's region from an internet search. We contacted these offices via telephone and asked to speak to an office manager or someone responsible for triaging patient phone queries. Using a scripted form, trained investigators questioned 100 consecutive primary care provider offices on how they would handle these poisoning-related calls if there was no poison center to refer their patients to. Results of our survey suggest that 82.5% of poisoning-related calls to primary care offices would be referred to 911 or an emergency department if there was no poison center. These results further support the role that poison centers play in patient care and health care utilization.

## 1. Background

In 2009 US poison control centers (PCCs) handled over 4.2 million calls related to poisonings, drug information, and environmental exposures (e.g., envenomations), including almost 2.5 million human exposures [1]. Over 90% of these exposures occurred at the caller's residence and 72.5% were managed on site, thereby not requiring an evaluation at a health care facility. These numbers of calls and percentage of on-site management have been consistent for several years [2].

 Past work has shown that PCCs can save health care resources [3–11] including the prevention of unnecessary emergency department (ED) visits, and decrease lengths of stay for poisoned patients [4, 12–14]. One study, involving 2007 data from our single PCC, showed a median savings of $33 million in unnecessary health care charges by managing patients at home [11]. A report from the United States Institute of Medicine estimates that the combined activities of all US PCCs save more than $900 million annually [15, 16]. Other reviews have identified the importance of maintaining government's financial support of PCCs, [15, 17] including one cost analysis that offered an appropriate summary by concluding that “poison control centers offer a large return on investment” [18]. Despite these data PCCs continue to be challenged with budget cuts [17, 19, 20].

Our PCC routinely conducts quality assurance surveys to identify our need and role within our community. We interview callers and health care providers on several issues including their satisfaction with our services, evolving needs, and alternative plans if our center were to close. We hypothesized that primary care providers (PCPs) would refer most poisoning-related calls to Emergency Medical Services (EMS), which we defined as 911 services or an ED, if our PCC were to close.

## 2. Method 

We conducted a cross-sectional telephone survey of PCP offices in our PCC's service region. Adult, family practice, and pediatric PCP offices were identified via an internet search, and phone numbers were recorded. We developed a scripted survey (see Appendix) that included an introductory statement (that identified the caller and the purpose of the call), three poisoning-related scenarios based on common calls to our PCC, and a fourth question related to after-hours calls. Trained investigators contacted PCP offices via telephone and asked to speak with an office manager or triage personnel who would handle a call from a patient or a patient's caregiver. After identifying an appropriate staff member, a structured phone interview was conducted. Each question was asked along with the four offered responses. All answers were recorded on a data abstraction sheet. All responses that did not fit into a predetermined answer were recorded under the category “other.” 

If the interview was not conducted on the first call, we left a message with a call back number. All offices not returning our message were called a second time. If there was no response after two calls or messages, the office was listed as a refusal to participate. The first 100 consecutively completed surveys were recorded and analyzed. Responses for discrete variables were totaled and reported as percentages. This was a quality assurance project that was exempt from IRB approval.

## 3. Results

**Figure 1:**
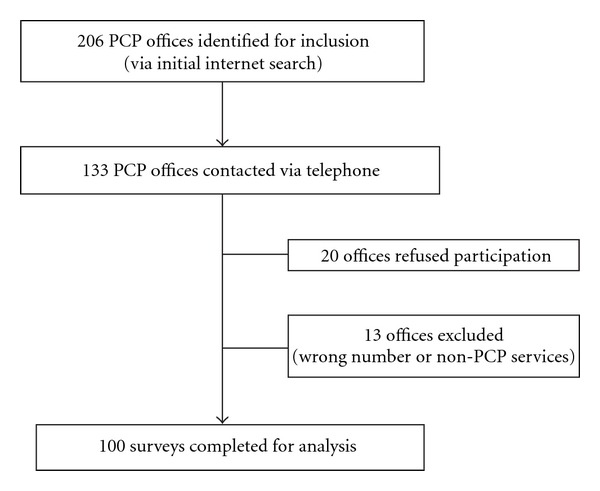
Survey data: selection and exclusion of PCP offices.

A total of 206 PCP offices were initially identified; 133 (64.5%) were contacted before 100 completed surveys were recorded. (Figure 1) Of the 33 offices not included, 20 (61%) refused participation and 13 (397%) were identified as non-PCP practices or had a nonworking telephone number. The survey results are represented in Tables 1 and 2. Overall, 82.5% of responses resulted in a patient referral to EMS (i.e., an ED or 911).

For the first question, “The patient (child) had an accidental ingestion of an unknown pill,” 92 subjects (92%) would refer the patient to an ED (*n* = 59) or 911 (*n* = 33). For the second question, “The patient (child) had an accidental exposure to fumes from an oven cleaner and was coughing with eye and throat irritation,” 90 subjects (90%) would refer the patient to an ED (*n* = 45) or 911 (*n* = 45). For the third question, “The patient (child) was stung on the foot by a scorpion and was crying with localized pain and paresthesias,” 70 subjects (70%) would refer the patient to an ED (*n* = 52) or 911 (*n* = 18).

Responses to the first three questions that were recorded in the “Other” category included “referral to an urgent care or pharmacy” (*n* = 3), “referral to urgent care” (*n* = 2), and “talk to the provider about advice” (*n* = 3).

For the fourth question, “Would there be a difference in handling these scenarios for after-hour calls,” 25 subjects (25%) said “no.” The 75 “yes” responses included “send to the ED” (*n* = 46), “page the oncall MD” (*n* = 17), “call 911” (*n* = 11), and “refer to a nurse line” (*n* = 1).

After combining the results for all questions, the overall rate of PCP referral of poisoning-related calls to EMS was 82.5%. This was determined by averaging the results of questions one through three (92%, 90%, and 70%, respectively), with the total EMS referrals for question four (84%). The total EMS referrals for question four was determined by adding the number of “yes” responses (that included refer to EMS; *n* = 57) with the number of “no” responses (“no” there would be no difference for after-hours calls) that represented a referral to EMS. To determine this number, we multiplied the “no” response (*n* = 25) by the average percentage of EMS referrals for questions one through three (84%), resulting in an additional 21 referrals. We then added this number of “no” responses (*n* = 21) to the number of “yes” responses (*n* = 57) and determined the final number of 78 (78%) for question four. Combining the percentages of EMS referrals for all four questions (no. 1 = 92%, no. 2 = 90%. no. 3 = 70% and no. 4 = 78%) resulted in an overall average of 82.5%.

## 4. Discussion

Previous work has tried to identify factors associated with healthcare utilization following poisoning-related illness, including patient or care-giver decisions and awareness of PCCs as a resource [3, 10]. Kearney et al. determined that 100% of surveyed EDs (*n* = 38) but only 82% of private physician offices (*n* = 114) would contact a PCC when dealing with a call from the public concerning a poisoning [3]. Of interest only 64% of the surveyed PCP offices in that study would refer a potentially poisoned patient to an ED, 911, or a physician office. It is unknown what percentage of these patients would ultimately be referred into the caller's PCP office instead of utilizing EMS? Factors associated with our increased rate of EMS referral (82.5% compared to, at most, 64%) are unknown, but may include increased private physicians' workload, increased fear of adverse patient outcome without immediate medical attention, or increased awareness of PCCs.

There are limited data concerning the involvement of PCCs for the care of patients admitted for poisoning-related illnesses. One study found that, overall, a regional PCC was consulted for only 18.1% of patients discharged after hospitalization for an accidental poisoning [4]. These authors found a higher PCC consultation rate for younger patients: 41.9% consultation rate for patients under six years of age compared to 6.7% for patients greater than 55 years of age. Using the National Electronic Injury Surveillance System, another study determined that a PCC was only contacted in about 19% of poisoning exposures, in patients under six years old, which resulted in an evaluation at a healthcare site [10].

Burkhart et al. evaluated the utilization of their regional PCC by ED and PCP physicians by using a 23-question survey [21]. With a response rate of nearly 40% (*n* = 715), these authors found that overall, 67% of the responding physicians had used PCC services within the past year. However, there was a much higher rate of PCC utilization among ED physicians (98.7%) compared to PCP physicians (65.3%). The reasons that these physicians called the PCC included assistance with poisoned patients (95%), poison education information (70%), identification of adverse drug reactions (46%), and drug identification (41%). Interestingly, despite overwhelming use of PCC services, responding ED physicians failed to report “approximately 50%” of their total poisoning cases.

Our data provide additional support of the role that poison centers play in saving health care resources. Through our role as a resource for PCP offices we help triage, and care for, patients with poisoning-related medical issues.

There are several limitations of this study, including the potential for selection bias and limited external validity. We included three poisoning-related scenarios based on our local experiences. These calls do not represent typical calls to PCCs, or PCP offices, in other areas of our country. Also, using a scripted survey, respondents were only offered a limited amount of patient information. It is possible that other decisions would have been made (e.g. home observation) if additional questions could have been asked and answered. In terms of selection bias, an office staff more supportive of the PCC may have been more likely to participate in the study. Offices with triage protocols or on-going staff education may feel more comfortable with handling poisoning-related issues and therefore may not have agreed to participate in the survey. Although we did not encounter any respondents that suggested this to be true, potential bias exists. Lastly, it is unknown how many of these fictitious patients, if referred to our PCC, would ultimately have been referred to EMS despite our involvement? However, during the study period our center managed 74% of human exposures onsite (e.g., at home) without referral to a health care facility.

## 5. Conclusions

Based on our survey, 82.5% of poisoning-related calls to primary care offices in our region would be referred to 911 or an ED if our poison center closed. These results support the role of poison control centers as a community asset by assisting with patient care and saving health care resources. 

## Figures and Tables

**Table 1:** PCP survey results; questions no. 1–3 (poisoning-related scenarios).

Survey questions 1–3	Responses to questions (*n* = 100)				
“Assuming there were no regional poison control centers”	Come to the office	Call 911	Go to the ED	Other	911 or ED
(1) The patient had an accidental ingestion of an unknown pill?	5	33	59	3	92
(2) The patient had an accidental exposure to fumes from an oven cleaner and has eye and throat irritation?	7	45	45	3	90
(3) The patient was stung on the foot by a scorpion and is having localized pain and paresthesias?	28	18	52	2	70

**Table 2:** PCP survey results; question no. 4 (after-hours calls).

Survey question 4	No	Yes
(4) Would there be a difference in handling these scenarios for after-hours calls?	25	75 (Send to the ED = 46; page oncall MD-17; call 911-11; refer to nurse line-1)

## Appendix

### Survey of PCP Offices regarding Poisoning-Related Issues

“Hello, my name is _________________ and I am calling on behalf of the Banner Good Samaritan Poison and Drug Information Center. I was hoping to speak with the office manager or any staff member who would triage a phone call from a patient. We have four short questions regarding poison center support and patient care that I would like to ask you. Assuming that the regional poison center was closed; if a patient or parent called in to your office with one of the following questions, what would you advise?”

1. The patient (child) had an accidental ingestion of an unknown pill?
  Ask patient to come into the office
  Instruct the patient to call 911
  Instruct the patient to go to the ED
  Other: _____________________
2. The patient (child) had an accidental exposure to fumes from an oven cleaner and was coughing with eye and throat irritation?
  Ask patient to come into the office
  Instruct the patient to call 911
  Instruct the patient to go to the ED
  Other: _____________________
3. The patient (child) was stung on the foot by a scorpion and was crying with localized pain and paresthesias?
  Ask patient to come into the office
  Instruct the patient to call 911
  Instruct the patient to go to the ED
  Other: _____________________.
4. Would there be a difference in handling these scenarios for after-hours calls?
  No
  Yes (if so how): _________________
